# Population prevalence of high dose paracetamol in dispensed paracetamol/opioid prescription combinations: an observational study

**DOI:** 10.1186/1472-6904-12-11

**Published:** 2012-06-18

**Authors:** Roderick Clark, Judith E Fisher, Ingrid S Sketris, Grace M Johnston

**Affiliations:** 1 Department of Community Health and Epidemiology, Dalhousie University, Halifax, NS, Canada; 2 College of Pharmacy, Faculty of Health Professions, Dalhousie University, Halifax, NS, Canada; 3 Pharmaceutical Services, Department of Health and Wellness, Halifax, NS, Canada; 4 School of Health Administration, Faculty of Health Professions Dalhousie University, Halifax, NS, Canada

## Abstract

**Background:**

Paracetamol (acetaminophen) is generally considered a safe medication, but is associated with hepatotoxicity at doses above doses of 4.0 g/day, and even below this daily dose in certain populations.

**Methods:**

The Nova Scotia Prescription Monitoring Program (NSPMP) in the Canadian province of Nova Scotia is a legislated organization that collects dispensing information on all out-of-hospital prescription controlled drugs dispensed for all Nova Scotia residents. The NSPMP provided data to track all paracetamol/opioids redeemed by adults in Nova Scotia, from July 1, 2005 to June 30, 2010. Trends in the number of adults dispensed these prescriptions and the numbers of prescriptions and tablets dispensed over this period were determined. The numbers and proportions of adults who filled prescriptions exceeding 4.0 g/day and 3.25 g/day were determined for the one-year period July 1, 2009 to June 30, 2010. Data were stratified by sex and age (<65 versus 65+).

**Results:**

Both the number of prescriptions filled and the number of tablets dispensed increased over the study period, although the proportion of the adult population who filled at least one paracetamol/opioid prescription was lower in each successive one-year period. From July 2009 to June 2010, one in 12 adults (n = 59,197) filled prescriptions for over 13 million paracetamol/opioid tablets. Six percent (n = 3,786) filled prescriptions that exceeded 4.0 g/day and 18.6% (n = 11,008) exceeded 3.25 g/day of paracetamol at least once. These findings exclude non-prescription paracetamol and paracetamol–only prescribed medications.

**Conclusions:**

A substantial number of individuals who redeem prescriptions for paracetamol/opioid combinations may be at risk of paracetamol-related hepatotoxicity. Healthcare professionals must be vigilant when prescribing and dispensing these medications in order to reduce the associated risks.

## Background

Paracetamol (acetaminophen) is a commonly used analgesic that has been considered safe at doses below 4.0 grams per day. [1-3] However, acute overdose [4], chronic doses over 4–6 g/day [5] and lower doses in certain populations [3,6-8], may be associated with hepatotoxicity. The Acute Liver Failure Study Group found that the median dose among American patients with unintentional overdose causing acute liver failure was 7.5 g per day, range of 1.0-78 g. [4] Paracetamol-related hepatotoxicity occurs through a complex sequence.[9,10] In high single doses (15 g or more), paracetamol causes hepatic injury through a toxic metabolite, NAPQI (N-acetyl-p-benzoquinone imine) [11,12]. Acetaminophen has been postulated to cause liver injury by mechanisms including glutathione depletion, oxidative stress and mitochondrial dysfunction leading to loss of adenosine triphosphate (ATP). Factors that induce cytochrome P-450, such as alcohol consumption and possibly, malnutrition, increase NAPQI synthesis and contribute to glutathione depletion, enhancing paracetamol-related hepatotoxicity [3,11,12].

Paracetamol-induced hepatotoxicity as a result of intentional or unintentional overdose is the most common drug-related cause of acute liver failure (ALF) in the USA, UK, Canada and most European countries, accounting for about one-half of all cases in the USA. [4,8,12-15] In the USA, about 150,000 poisoning cases were attributed to paracetamol in 2009, according to the Annual Report of the American Association of Poison Control Center’s National Poison Data System. [16] An estimated 70,000 cases occur annually in the UK [17,18]; in Canada, the estimated annual incidence of paracetamol overdose between 1997 and 2002 was about 46 per 100,000 population[13]. A substantial proportion of these cases may be unintentional or ‘therapeutic misadventures’. Two American studies report that respectively one-half [4] and two-thirds [19] of identified paracetamol-related overdose cases were unintentional. In 2011 in Nova Scotia, there were 62 calls to the Nova Scotia Poison Centre with unintentional paracetamol (or paracetamol combination product) poisonings in people over 18 years of age. Of these, 23 were paracetamol/opioid combination products (Kim Sheppard R.N., B.ScN., CSPI. Clinical Leader IWK Regional Poison Centre. Personal Communication, April 13^th^ 2012). Paracetamol toxicity can be difficult to diagnose, however; one study suggests that 18% of indeterminate cases of liver failure referred to an American tertiary care centre were due to unrecognized paracetamol toxicity [20].

A large number of paracetamol-containing products are available as both non-prescription and prescription medications. For example, as of August 2011, there were 434 paracetamol-containing medications available on the Canadian market.[21] Non-prescription products include cough and cold preparations, and analgesics and antipyretics. The high rates of paracetamol use may be due in part to recommendations that persons using acetylsalicylic acid for arthritis or other painful conditions consider taking paracetamol, to reduce the potential for gastrointestinal side effects [22].

Prescription medications include combinations with opioid analgesics such as hydrocodone, oxycodone and codeine. Paracetamol/opioid compounds are implicated in a substantial proportion of paracetamol-induced hepatotoxicity cases.[4,23] For example, an American study found that 44% of individuals in a cohort of 275 consecutive patients with paracetamol-related ALF reported taking a prescription paracetamol/opioid combination.[4] These medications are very commonly prescribed in both the USA and Canada. In 2010, 131 million prescriptions for acetaminophen (paracetamol) in combination with hydrocodone were filled in the USA, making this medication the most commonly dispensed prescription drug.[24] This combination is not available in Canada; however, Canadians filled about 8.3 million prescriptions in 2010 for paracetamol/opioid compounds, such as acetaminophen (paracetamol)/caffeine/codeine combinations (~5.5 million) and acetaminophen (paracetamol)/oxycodone (~2.7 million).[25] Because these products are fixed dose combinations, increasing the opioid dose results in an increase in the paracetamol dose also. Therefore, such fixed dose combinations may not be appropriate for all patients; for some patients, the paracetamol and opioid should be titrated separately. [26] Further, the relative contribution of the opioid to the hepatotoxicity is unknown [27].

Professional and public education initiatives and legislation to limit paracetamol use are increasingly being suggested, in order to limit the potential harm from overuse of paracetamol. [20,28-30] However, only a few population level studies, all using American data, [31,32] have documented the prevalence of paracetamol use exceeding the dosage limits recommended by regulatory bodies. The objective of this study is to provide population-based Canadian data on the prevalence of high-dose paracetamol use from prescribed paracetamol/opioid combinations.

## Methods

Data were extracted from the electronic database of the Nova Scotia Prescription Monitoring Program (NSPMP). Paracetamol/opioid combinations are “controlled substances” under Canadian federal [33] and provincial legislation. In the province of Nova Scotia, the monitoring of prescribed controlled substances dispensed in the community is the legislated responsibility of the NSPMP.[34,35] The NSPMP collects dispensing information on all controlled drugs for all people dispensed in NS community pharmacies within an electronic database, and reviews and investigates use patterns that suggest potentially inappropriate use.[34,35] This database does not include medications dispensed while a person is in hospital, but it does include medications prescribed for nursing home residents.

The study population included all Nova Scotia residents age 19+ years eligible for provincial health benefits who were dispensed any paracetamol/opioid combination (World Health Organization (WHO) Anatomical Therapeutic Chemical (ATC) Classification: N02AA59, N02BE51)[36] in community pharmacies from July 1, 2005 to June 30, 2010. In Nova Scotia, virtually all residents are eligible for provincial health benefits.[37] The adult population of Nova Scotia increased from 751,000 to 768,000 over this period.[38]

For each one-year period, July 1, 2005 to June 30, 2010, three totals were extracted: individuals filling at least one paracetamol/opioid prescription; paracetamol/opioid prescriptions dispensed; and tablets dispensed. The trend over time (2005–2010) was examined using the Cochran-Armitage test [39].

The average paracetamol daily dose was calculated for each individual who filled a paracetamol/opioid prescription from July 1, 2009 to June 30, 2010 (mg paracetamol per tablet X quantity of tablets dispensed/days’ supply). These products contain either 300 mg or 325 mg paracetamol per tablet.[21] Provincial regulations [40] require prescribers to include the intended days’ supply on the prescription. The numbers of individuals who filled prescriptions supplying average daily doses exceeding 3.25 g and 4.0 g during this period at least once and more than once were determined.

The data were stratified by sex and age (<65 years, 65+). Differences by sex and age for each one-year period were examined using chi square tests with Microsoft Excel 2010^TM^™.

Dalhousie Health Sciences Ethics Review Board approved the research protocol.

## Results

**Figure 1:**
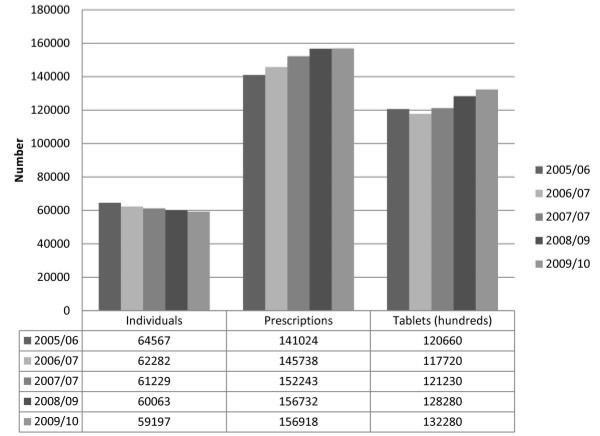
The number of adult (age 19+) Nova Scotia residents who filled at least one paracetamol/opioid prescription, prescriptions filled and tablets dispensed (in hundreds) in each one-year period from July 1, 2005 to June 30, 2010.

**Figure 2:**
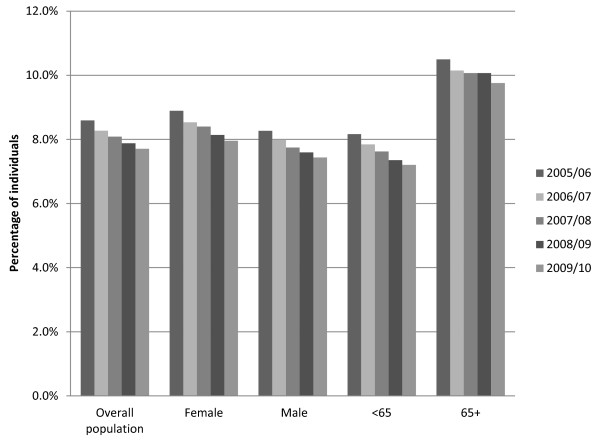
**Percentage of adult (age 19+) Nova Scotia residents who filled at least one prescription for paracetamol/opioid, by sex and age, and overall, per one-year period: July 1, 2005 to June 30, 2010.** Denominator is adult population of Nova Scotia for each year, based on data from Statistics Canada.

Both the number of prescriptions filled and the number of tablets dispensed increased over the study period (Figure 1). Tests for trend showed an annual increase from July 2005 through June 2010 in the total prescriptions by age and sex and in total tablets by age. However, as shown in Figures 1 and 2, the number of individuals and the proportion of the adult population who filled at least one paracetamol/opioid prescription was lower in each successive one-year period from 2005 to 2010 [e.g. 64,567 (8.6%) in 2005/06 versus 59,197 (7.7%) in 2009/10].

In each one-year period, a greater proportion of women than men filled at least one prescription for paracetamol/opioid combinations [e.g. 8.9% versus. 8.3% in 2005/06, and 8.0% versus 7.4% in 2009/10, p < .001]. A greater proportion of individuals age 65 and older, than those younger than 65, filled at least one prescription.

Among the 7.7% of adult Nova Scotians who filled at least one prescription for paracetamol/opioids in 2009/10, 18.6% (n = 11,008) filled prescriptions providing an average daily paracetamol dose over 3.25 g at least once and 6.4% (n = 3,786) over 4.0 g/day (Table 1). Individuals exceeded these respective dose limits more than once at rates of 21% (n = 2,307) and 10% (n = 395), respectively.

**Table 1:** **Number and Percent of adult (age 19+) Nova Scotia residents who fill at least one prescription for an acetaminophen/opioid product and whose prescriptions are filled for over 3.25 g/day and 4.0 g/day (July 1**^**st**^**2009 to June 30**^**th**^**2010)**

		Individuals who filled at least one prescription	Average prescriptions per individual^1^	> 3.25 g/day(%)	> 4.0 g/day(%)
	Male	27,384	2.53	4,939 (18 %)	2,145(7.8%)
Sex					
	Female	31,813	2.80	6,069 (19.0%)	1,641 (5.1%)
	<65	44,443	2.62	8,216 (18.4%)	3,062 (6.8%)
Age					
	65+	14,754	2.75	2,792 (18.9%)	724 (4.9%)
Total		59,197	2.68	11,008 (18.6 %)	3,786 (6.4 %)

1. “Average prescriptions” was computed based on the total number of individuals who filled at least one prescription divided by the total number of prescriptions dispensed during this time period.

2. The denominator for the percentage is individuals who filled at least one prescription during this time period.

A greater proportion of women than men filled prescriptions exceeding 4.0 g/day [6.7% versus 6.0%, p < .001] and 3.25 g/day [19.1% versus 18.0%, p = .001] at least once during this one-year period. A larger percentage of women than men exceeded 3.25 g/day more than once [26.1% versus 14.6%, p < .001].

Compared with those age 65 and older, a greater proportion of younger individuals filled prescriptions exceeding 4.0 g/day [6.9% versus 4.9%, p < .001] at least once. However, a similar proportion of younger and older individuals filled prescriptions exceeding 3.25 g/day [18.5% versus 18.9%, ns]. A larger percentage of those age 65 and older filled prescriptions exceeding these daily limits more than once [14.2% (65+) versus 9.5% (<65), p < .001 (4.0 g/day); 25.9% versus 19.3%, p < .001 (3.25 g/day)].

## Discussion

Of the approximately 760,000 adults in Nova Scotia from 2005–2009, almost 60,000 (7.9%) adults, redeemed an out-of-hospital prescription for paracetamol/opioid combinations over a one-year period (July 2009/2010). Of this 60,000, almost 4,000 (6.7%) filled prescriptions at least once that provided daily doses exceeding the usual Health Canada recommended maximum (4.0 g). This finding is consistent with the findings of Mort et al. [41] and Albertson et al. [32] who report rates of 8.1% and 5.9% respectively, but lower than the rate (23.3%) observed in other studies [31]. The Nova Scotia and other study findings are considered underestimates because they exclude medications dispensed in-hospital, non-prescription paracetamol containing products and paracetamol–only prescriptions. However, an advantage of the Nova Scotia study over previously published population studies is that this study includes virtually all persons in a geographic area and is not limited by the enrollment criteria of US-based health insurance programs.

One in five, more than 10,000 individuals, filled at least one prescription with a daily paracetamol dose greater than 3.25 g. Further, the number and percentage of adults redeeming out-of-hospital prescriptions for paracetamol/opioid combinations decreased in each year from 2005 through 2010, despite a slight population increase, but the numbers of prescriptions filled and tablets dispensed increased. This finding suggests that fewer individuals filled more prescriptions for more tablets over this period. Taken together, these findings raise concerns regarding the potential for a substantial number of adults to be at risk for paracetamol-induced hepatotoxicity due to the unintentional ingestion of high doses of paracetamol.

Certain sub-populations may be particularly vulnerable to accidental overdose. The findings suggest that older persons, i.e. those age 65 and over, may fill more prescriptions for these combinations than younger persons, and for larger quantities of tablets. Older persons may be particularly likely to consume paracetamol-containing medications long-term because of their high prevalence of painful conditions such as osteoarthritis.[42] The finding that among those who filled prescriptions exceeding 4.0 g/day or 3.25 g/day, older persons were significantly more likely to do so multiple times raises concerns that they may be at increased risk of chronically consuming high-dose paracetamol.

Similarly, women may be at higher risk than men, given that a greater percentage of women than men filled any prescription and filled prescriptions that exceeded 4.0 g/day (7%) and 3.25 g/day (19%). Li and Martin [2011] observed a higher rate of paracetamol overdose among females presenting in emergency departments that provide care to children, youth and adults. [43] The authors speculated that compared with males, females use more non-prescription analgesics for longer durations, and are more likely to use these medications in suicide attempts.[43] In contrast, Mort et al. [41] observed a significantly higher rate of paracetamol use among male beneficiaries of three insurance programs. The possibility of an interaction between sex and age was not examined in any of these studies, and the population characteristics varied markedly.

More than one-quarter of women and one-quarter of persons age 65 and over who filled prescriptions that provided more than 3.25 g/day did so multiple times. These patterns of use, together with the widespread availability and use of non-prescribed paracetamol-containing products raise the potential for the unintentional consumption of high – and potentially hepatotoxic – doses of paracetamol. Individuals may take paracetamol-containing products such as a cough and cold preparation or plain non-prescription paracetamol in addition to their prescribed analgesic and be unaware of the actual amount of paracetamol that they are consuming.

The use of paracetamol/opioid compounds may place patients at increased risk of unintentional paracetamol overdose because they are fixed-dose combinations. Increasing the opioid dose – in order to achieve adequate analgesia - also increases the dose of paracetamol, possibly over the recommended daily maximum dose. In Canada, there is a high consumption of codeine-paracetamol compounds. [44] The analgesic efficacy of codeine is potentially unreliable because of its unpredictable pharmacokinetics. [45,46] Codeine is a prodrug that must be converted to morphine. This conversion depends on the polymorphic cytochrome P4502D6 (CYP2D6) pathway. Genetic polymorphisms result in three phenotypes, poor, extensive and ultra-rapid metabolizers. A poor metabolizer may receive almost no analgesic effect from a standard dose of codeine. [47,48] Prescribers need to evaluate the role of these compounds compared with other analgesics and determine the risks and benefits for individual patients.

An American, multicentre, prospective study of 275 identified paracetamol-induced cases of acute liver failure, 48% of cases were unintentional or ‘therapeutic misadventures’.[4] Most (79%) of these individuals reported taking paracetamol for pain, almost two-thirds (63%) reported taking a prescription paracetamol/opioid combination and 38% were consuming two paracetamol-containing products concurrently. Of particular concern was the fact that these persons have poorer outcomes than those with intentional overdoses; a significantly greater percentage presented with severe (grades 3 and 4) hepatic encephalopathy, possibly because of a delay in seeking medical care.[4] Two Canadian studies found lower proportions of unintentional overdoses, 25% [14] and 13% [13] respectively. However, while paracetamol overdose-related hospitalisations declined from 1995 to 2004 among persons younger than 50 years, the rate increased for those ages 50 and over, as did their rate of hospitalisation.

Various strategies have been proposed or implemented in an effort to reduce paracetamol-related harms. The UK introduced legislation that limits the quantity of paracetamol sold without prescription and required these products to be blister-packed. In 2009, the US Food and Drug Administration (FDA) convened an internal working group from the Center for Drug Evaluation and Research (CDER) to prepare a report on the issue of paracetamol-related hepatotoxicity in preparation for the joint meeting of three Advisory Committees: Drug Safety and Risk Management; Nonprescription Drug; and Anesthetic and Life Support Drugs.[29,30,49] Upon discussion of the working group’s report, the Advisory Committees accepted some, but not all, of the working group’s recommendations. The Committees recommended: the elimination of prescription paracetamol combinations; reduction of the maximum daily dose to 3.25 g and the maximum individual dose to 650 mg; designating 500 mg tablets as prescription-only; and a single concentration for all liquid products. The Committees also voted to encourage the FDA to promote awareness among health professionals by including a black box warning in product information and to encourage patient-education regarding the potential risks.[29,30,49] In Canada, the National Opioid Guideline Group recommends that paracetamol doses not exceed 3.20 g/day for the management of chronic non-cancer pain.[50,51] Health Canada issued an advisory in January 2011 reminding Canadians about using paracetamol wisely [52] and referencing the labelling requirements for over-the-counter products that include a maximum daily dose of 4.0 g for adults and children 12 years and older [2].

Healthcare professionals must be vigilant when prescribing and dispensing paracetamol/opioid combinations and educate their patients about paracetamol-containing products and their potential toxicity. The need for greater public education has been recognized.[41] Persons who receive care from multiple health care prescribers such as physicians (primary care and specialists), dentists, pharmacists and nurse practitioners may be particularly at risk. In the future, computer generated alerts in decision support systems might be helpful in determining maximum doses for over-the-counter and prescription paracetamol-containing medications.

A limitation to our study is that patient level information was not available. For example, data were not available regarding patient weight, ethnicity, type and severity of pain or adequacy of pain control, comorbidities, patient preferences or specific risk factors for hepatotoxicity such as the presence of non-alcoholic fatty liver disease, or the concomitant use of other drugs or herbal therapies.[3,7,32,53-56] In addition, we were unable to determine the rates of genetic polymorphisms of the CYP2D6 pathway in our population.[45,46] Further, the extent to which the population was at a palliative stage is unknown; stratified analysis by this time period of need for increased pain management is warranted.

Research is required to provide a more extensive understanding of the number of high risk individuals, treatment appropriateness and methods employed to optimize treatment, and the relationship between therapeutic doses of paracetamol and acute liver failure. In addition, investigation of the sex-based differences in patterns of use is warranted. Further, as government regulators and clinical practice guidelines adopt lower maximum doses, it will be important to determine if the application of these guidelines results in increased use of non-steroidal anti-inflammatory drugs (NSAIDS) and increased NSAID-related adverse events [28].

Comprehensive evidence on the extent of paracetamol use and the relationship between therapeutic doses of paracteamol and acute liver failure is sparse as yet, as is the value of interventions that attempt to reduce harm [57]. Further population-based studies are needed to better understand the role of paracetamol in the etiology of acute liver failure and to monitor changes over time as multi-faceted interventions are introduced in an attempt to limit harm from pain medications.

## Conclusions

About one in 12 adults in Nova Scotia filled at least one prescription for paracetamol/opioid combination drugs during a one-year period starting July 2009. Of these individuals, six percent filled prescriptions that supply paracetamol doses greater than the usual recommended daily dose of 4.0 g and one in five exceeded 3.25 g/day. These individuals may be at risk for paracetamol-related hepatotoxicity. Given the widespread use of paracetamol-containing products, including non-prescription products, the potential exists for unintentional paracetamol overdose. Health professionals, policy makers, patients and caregivers must be made aware of the many products containing paracetamol, their potential for toxicity, and approaches to minimize the risks.

## Competing interests

Dr. Ingrid Sketris has received compensation from Green Shield Canada and Health Canada. Other authors declare they have no competing interests.

## Authors’ contributions

JF, RC and IS contributed to conception and design, JF and RC in data analysis and all authors participated in interpretation of the data and drafting or critical review of the manuscript. All authors read and approved the final manuscript.

## Pre-publication history

The pre-publication history for this paper can be accessed here:

http://www.biomedcentral.com/1472-6904/12/11/prepub
